# BET protein inhibitor JQ1 downregulates chromatin accessibility and suppresses metastasis of gastric cancer via inactivating RUNX2/NID1 signaling

**DOI:** 10.1038/s41389-020-0218-z

**Published:** 2020-03-10

**Authors:** Siqi Zhou, Shu Zhang, Lei Wang, Shuling Huang, Yue Yuan, Jie Yang, Hui Wang, Xihan Li, Pin Wang, Lin Zhou, Jun Yang, Yuemei Xu, Huan Gao, Yixuan Zhang, Ying Lv, Xiaoping Zou

**Affiliations:** 1 Department of Gastroenterology, Nanjing Medical University Affiliated Drum Tower Clinical Medical College, Nanjing, 210008 China; 2 Jiangsu Clinical Medical Center of Digestive Diseases, Nanjing, Jiangsu China; 3 Department of Gastroenterology, Drum Tower Hospital Affiliated to Medical School of Nanjing University, Nanjing, 210008 China; 4 Department of Pathology, Drum Tower Hospital Affiliated to Medical School of Nanjing University, Nanjing, 210008 China; 5 Life Science Department, Vazyme Biotech Co., Nanjing State Economy and Technology Development Zone, Nanjing, 210000 China

**Keywords:** Gastric cancer, Cell migration

## Abstract

Chromatin accessibility is critical for tumor development, whose mechanisms remain unclear. As a crucial regulator for chromatin remodeling, BRD4 promotes tumor progression by regulating multiple genes. As a small-molecule inhibitor of BRD4, JQ1 has potent chemotherapeutic activity against various human cancers. However, whether JQ1 has potential anti-tumor effects and how JQ1 regulates global transcription in gastric cancer (GC) remain largely unknown. In this research, we found BRD4 was highly expressed in GC tissues and was significantly associated with poor prognosis. JQ1 inhibited the proliferation and induced apoptosis of GC cells in vitro. Besides, JQ1 suppressed the migration and invasion of cancer cells by inducing MET. Notably, an assay for transposase-accessible chromatin using sequencing (ATAC-seq) data showed that JQ1 obviously downregulated the chromatin accessibility of GC cells and differentially accessible regions were highly enriched for RUNX2-binding motifs. Combinational analysis of ATAC-seq and RNA-seq data discovered NID1 as the downstream target of JQ1 and JQ1 reduced NID1 expression in GC cells. Chromatin immunoprecipitation, luciferase reporter gene assay, and rescue experiments all confirmed that RUNX2/NID1 axis was responsible for JQ1-inhibiting metastasis of GC cells. What’s more, high expression of NID1 in GC tissues also predicted poor survival outcome of cancer patients and NID1 knockdown prohibited migration and invasion of cancer cells via partially inducing MET. Finally, in vivo models showed that JQ1 prevented GC growth and suppressed cancer metastasis. In conclusion, JQ1 inhibits the malignant progression of GC by downregulating chromatin accessibility and inactivating RUNX2/NID1 signaling. In addition, NID1 is also a novel therapeutic target for progressive GC patients.

## Introduction

Gastric cancer (GC) is an aggressive disease whose prognosis is still poor and, generally, has no specific symptoms that renders early diagnosis of this disease difficult^1^. Although various treatments have been developed, many of them have failed to cure metastatic and progressive GC patients^2^. Thus, it is urgent to explore novel diagnostic markers and molecular mechanisms responsible for the progression of GC.

Chromatin accessibility has been identified as one of the most relevant genomic characteristics associated with oncologic functions at a specific site, providing the structure for transcription factor (TF) binding to regulate multiple genes correlated to cancer progression and invasion^3^. Recently, many researchers have started to investigate into how chromatin-state changes affect cancer development^4^. Dysregulated chromatin accessibility might alter the transcription activities of downstream oncogenes or tumor suppressor genes, thus driving malignant progression^5^. Histone acetylation and deacetylation are important epigenetic processes regulating gene expression via chromatin remodeling. Adding acetyl groups to lysine residues of histone proteins by histone acetyltransferases weakens polar interaction between histone proteins and DNA, relaxes chromatin structure, and drives genes more easily for transcription^6^. The bromodomain and extraterminal domain (BET) family contains proteins, such BRD3 and BRD4, capable of recognizing acetylated histone lysine residues. By accumulating on hyperacetylated chromatin regions as active promoters or enhancers, these proteins serve as scaffolds for recruiting TFs and multi-protein complexes that promote transcription of target genes^7^, thus possibly changing global chromatin accessibility. Dysregulation of these BET family proteins is commonly found in a variety of diseases including cancer^6^. As a result, they are potential therapeutic targets in the development of novel anticancer drugs^8^.

The small-molecule BET inhibitor JQ1 masks bromodomain acetyl-lysine-binding pockets and is highly specific for BET family proteins, especially bromodomain containing 4 (BRD4)^9^. JQ1 has effective chemotherapeutic activities against many hematological malignancies, such as acute myeloid leukemia^10^, multiple myeloma^11^, and acute lymphoblastic leukemia^12^. JQ1 also exerts anti-cancer effects against several solid tumors, including NUT midline carcinoma^13^, lung adenocarcinoma^14^, neuroblastoma^15^, and medulloblastoma^16^, mainly by inhibiting c-MYC functions^17^. Interestingly, more and more studies show that inhibition of c-MYC is not always the main mechanism of JQ1 in cancer cells^8^. Recently, researchers demonstrate that JQ1 suppresses GC growth by inducing cell apoptosis or senescence through regulating c-MYC or E2F/p21 signaling^18^. However, whether JQ1 has therapeutic effects on metastatic GC and how JQ1 regulates global transcription in GC cells still remain largely unknown.

In this study, we explored the potential role of JQ1 in inhibiting GC progression and metastasis. To investigate the relationship between chromatin changes and the transcriptional response to JQ1 in GC cells, we measured genome-wide chromatin accessibility (an assay for transposase-accessible chromatin using sequencing, ATAC-seq) and expression (RNA-sequencing, RNA-seq) changes resulting from JQ1 treatment. We identified a novel runt-related TF-2 (RUNX2)/Nidogen-1 (NID1) axis responsible for JQ1-induced suppression of GC cell metastasis. Our results suggest that BET protein inhibitor JQ1 prohibits the malignant progression of GC cells by downregulating chromatin accessibility and inactivating RUNX2/NID1 signaling. The clinical use of JQ1 in progressive GC patients deserves further investigations.

## Results

### Overexpression of BRD4 in GC tissues correlated with poor overall survival outcome

**Fig. 1:**
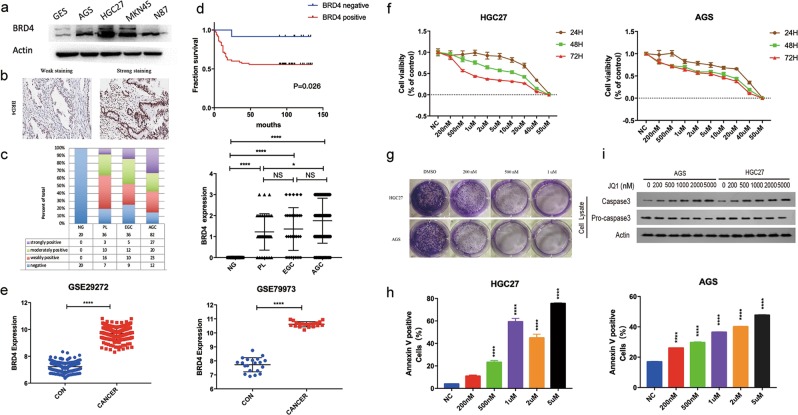
Expression of BRD4 in human GC cell lines and tissues, and the effects of JQ1 on GC cell proliferation and apoptosis. **a** The protein expression of BRD4 in GES-1 and various GC cell lines. **b** Immunohistochemistry staining showing weak expression and strong expression of BRD4 in the tumor tissues from two patients with AGC. **c** Differential expression of BRD4 in patients with AGC, EGC, and PL compared with NG (mean ± SEM, NS > 0.05, *p < 0.05, ****p < 0.0001). **d** Kaplan–Meier curves comparing overall survival in AGC patients with negative and positive expression of BRD4 (*n* = 82; *P* = 0.026, log-rank test). **e** GEPIA (Gene Expression Profiling Interactive Analysis) data from the GEO databases (dataset 29,272 and 79,973) demonstrates elevated mRNA levels of BRD4 in GC, compared with non-tumor tissues (mean ± SEM, ****p < 0.0001). **f** The proliferation rates of HGC27 and AGS cells treated with JQ1 (0, 200 nM, 500 nM, 1 μM, 2 μM, and 5 μM) for 72 h determined by CCK8 assay. **g** Colony-formation assay of HGC27 and AGS cells treated with JQ1 (200 nM, 500 nM, and 1 μM). **h** Flow cytometry analysis of the apoptotic cells treated with JQ1 (0, 200 nM, 500 nM, 1 μM, 2 μM, 5 μM) for 72 h using the Annexin V/PI staining assay. Positive Annexin V cells were displayed as histogram (mean ± SEM, ****p < 0.0001 compared with control cells). **i** WB analysis of the expression of caspase-3 and pro-caspase-3 in HGC27 and AGS cells treated with JQ1 (0, 200 nM, 500 nM, 1 μM, 2 μM, and 5 μM) for 72 h.

We first measured the protein expression of BRD4 in normal gastric epithelial cell line GES and some GC cell lines we owned in our laboratory. As shown in Fig. 1a, BRD4 expression in GC cell lines was much higher than that in GES. Next, we examined BRD4 expression in tissue samples from gastric precancerous lesions (PL/36), early GC (EGC/36), and advanced GC (AGC /82), as well as noncancerous gastric mucosa (NG/20) using immunohistochemistry (Fig. 1b). All the 20 NG samples stained negative for BRD4 antibody. Among the 36 PL samples, 80.6% of the patients exhibited weakly to strongly positive staining for BRD4, and only 8.3% of the PL patients showed strongly positive expression of BRD4. The positive rate of BRD4 expression in EGC patients turned out to be 75%. Lastly, 85.4% of the AGC patients stained positive for BRD4, among whom 32.9% exhibited strongly positive expression. Statistical analysis showed that BRD4 expression in AGC, EGC, and PL tissues was significantly higher than that in NG tissues, whereas BRD4 expression in AGC was obviously higher than in PL, suggesting that BRD4 might play an important role in the development of GC. (Fig. 1c) In addition, Kaplan–Meier (KM) survival analysis showed that positive expression of BRD4 was significantly associated with a poor survival outcome in AGC patients (*P* = 0.026) (Fig. 1d). Moreover, BRD4 mRNA expression was significantly higher in GC compared with the surrounding non-tumor gastric tissues in GEO GSE29272 and GSE79973 datasets (Fig. 1e). Taken together, these data indicated that BRD4 protein and mRNA levels were upregulated in human GC, and might be a potential prognostic biomarker for GC patients, thus providing a therapeutic rationale by targeting BRD4 in GC.

### Effects of JQ1 treatment on GC cell proliferation and apoptosis

As a BET inhibitor, JQ1 has been proved to exert anti-cancer effects against many hematological malignancies and solid tumors. Therefore, we investigated whether JQ1 had chemotherapeutic activities in GC cells. Based on expression levels, we chose AGS, HGC27, and MKN45 with high BRD4 expression for further experiments. Cell Counting Kit-8 (CCK8) assays revealed significant inhibition of cell proliferation in a dose-dependent manner in all the GC cell lines treated with JQ1 (Fig. 1f and Supplementary Fig. 1a). In accordance with the CCK8 results, JQ1 also decreased the colony-formation capacities of the GC cells dose-dependently (Fig. 1g). Next, we investigated the potential apoptotic effect of JQ1 using fluorescence-activated cell sorting assay. As shown in Fig. 1h and Supplementary Fig. 1b, JQ1 induced obviously increased apoptosis in GC cells. Consistent with the observation, western blotting (WB) analysis demonstrated upregulation of caspase-3 and downregulation of pro-caspase-3 levels in JQ1-treated GC cells. (Fig. 1i) These data suggest that JQ1 inhibits GC cell proliferation at least partially through induction of cellular apoptosis.

### JQ1 treatment suppresses GC cell migration and invasion via inducing MET both in vitro and in vivo

**Fig. 2:**
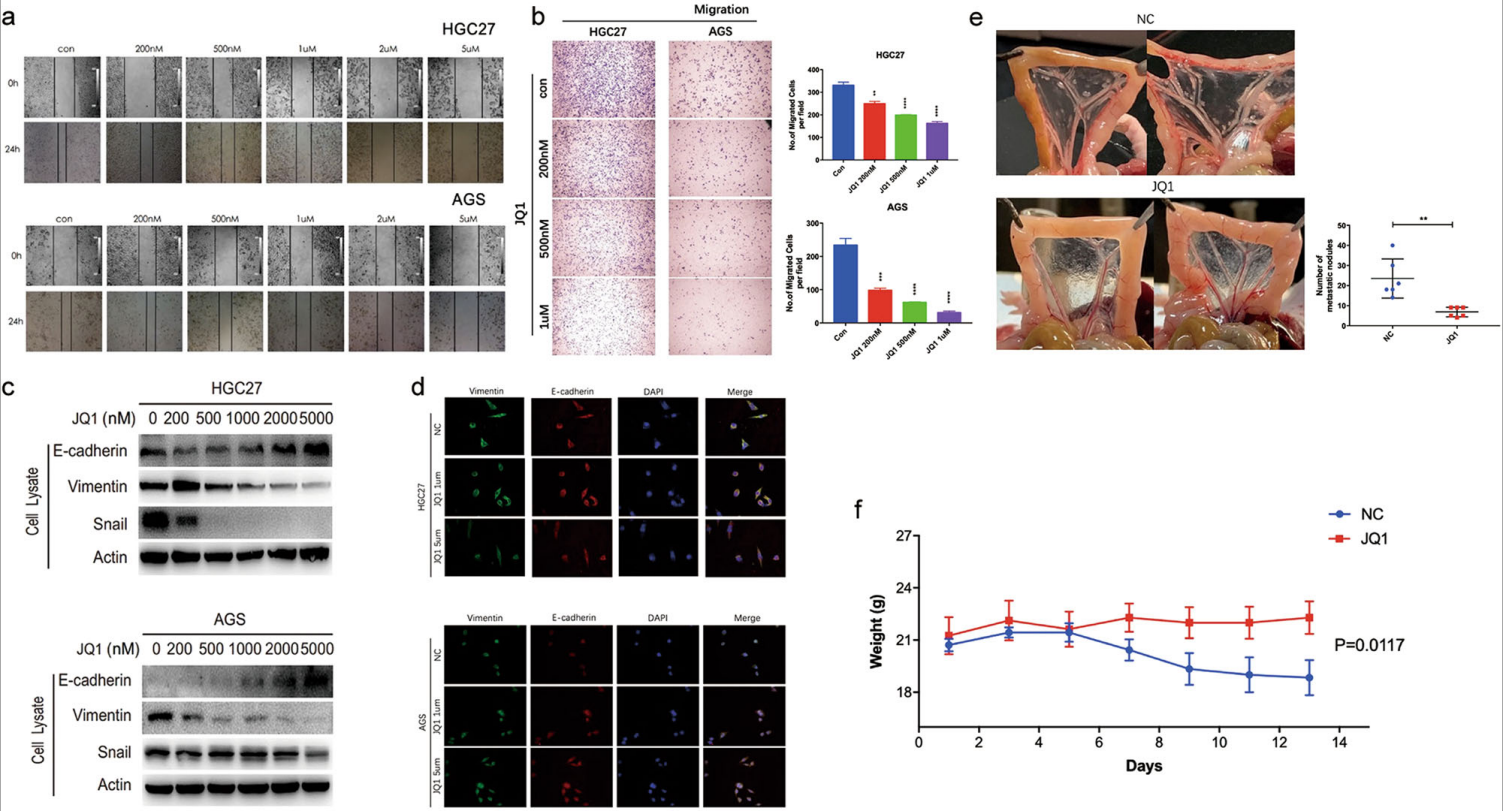
JQ1 treatment suppresses GC cell migration and invasion via inducing MET both in vitro and in vivo. **a** The migratory capability of HGC27 and AGS cells treated with JQ1 (0, 200 nM, 500 nM, 1 μM, 2 μM, and 5 μM) for 72 h revealed by wound-healing assay. **b** The number of migrating and invading GC cells after treatment with JQ1 (0, 200 nM, 500 nM, 1 μM, 2 μM, and 5 μM) for 72 h by transwell assays. Representative images of migrated and invaded HGC27 and AGS cells in each group were shown on the left. Cells that migrated and invaded through the pores of transwell plates were counted in five random fields and were reported on the right (mean ± SEM, **p < 0.01, ***p < 0.001, ****p < 0.0001). **c** WB analysis of the expression of E-cadherin, Vimentin, and Snail in HGC27 and AGS cells after treatment with JQ1 (0, 200 nM, 500 nM, 1 μM, 2 μM, and 5 μM) for 72 h. **d** Immunofluoresence staining of the EMT markers Vimentin and E-cadherin in HGC27 and AGS cells after JQ1 (0, 1 μM, and 5 μM) treatment. **e** The macroscopic nodules in the peritoneal cavity in each group of mice (*n* = 6) were photographed. The number of the macroscopic nodules in each mouse was recorded (mean ± SEM, **p < 0.01). **f** The body weights of each mouse were recorded (mean ± SEM, *p* = 0.0117).

**Fig. 3:**
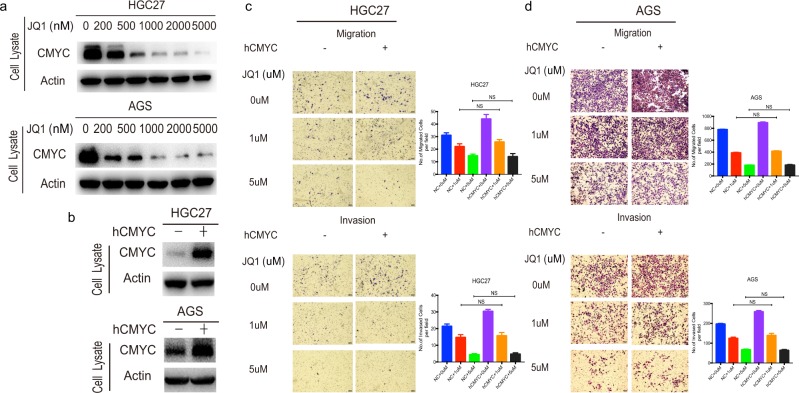
JQ1-inducing inhibition of GC cell metastasis is independent of c-MYC signaling. **a** The protein expression of c-MYC in HGC27 and AGS cells treated with JQ1 (0, 200 nM, 500 nM, 1 μM, 2 μM, and 5 μM) for 72 h shown by WB analysis. **b** The protein expression of c-MYC in HGC27 and AGS cells transfected with scrambled pcDNA3.1 and pcDNA3.1-c-MYC plasmids shown by WB analysis. **c**, **d** c-MYC overexpression can not antagonize the upregulation of the migration and invasion induced by JQ1 treatment in HGC27 and AGS cells. Representative images of migrated and invaded HGC27 and AGS cells in each group were shown on the left. Cells that migrated and invaded through the pores of transwell plates were counted in five random fields and were reported on the right. Data were presented as mean ± SEM of three independent experiments.

Next, we explored the effects of JQ1 treatment on cell migration and invasion in GC cell lines. Wound-healing assay showed that JQ1 significantly inhibited the migratory capability of GC cells in a dose-dependent manner in comparison with control cells (Fig. 2a and Supplementary Fig. 1c). Consistently, transwell assay exhibited a significant decrease of the number of migrating and invading cells in a dose-dependent manner in GC cells treated with JQ1 (Figs. 2b and 3c, d). Epithelial-to-mesenchymal transition (EMT) is a leading factor accounting for the metastatic ability of cancer cells. To investigate whether JQ1 treatment results in the reversion of EMT, we checked the protein expression of epithelial and mesenchymal markers. WB showed that JQ1 led to a dramatic upregulation of E-cadherin and concurrent downregulation of Vimentin and Snail expression levels both in AGS and HGC27 cells dose-dependently (Fig. 2c). Accordingly, immunofluoresence staining demonstrated decreased expression of Vimentin and increased expression of E-cadherin in GC cells after JQ1 treatment compared with control cells, suggesting that JQ1 suppressed GC cell migration and invasion possibly through inducing MET process (Fig. 2d).

To determine the therapeutic effect of JQ1 for metastatic GC, we performed an in vivo experiment by injecting HGC27 cells into the intraperitoneal (i.p.) cavities of nude mice. Then the mice were killed after 2 weeks’ treatment of JQ1 or vehicle control. The results showed that the mice treated with JQ1 exhibited reduced number of macroscopic nodules in the peritoneal cavity (Fig. 2e). Moreover, the body weight of the mice was heavier in JQ1-treating group than in the control group, suggesting that the metastasis-associated cachexia was alleviated by JQ1 (Fig. 2f). Collectively, JQ1 has great potential in inhibiting GC metastasis and suppressing malignant progression.

### JQ1-inducing inhibition of GC cell metastasis is independent of c-MYC signaling

To gain additional insights into the mechanism of JQ1, we first focused our attention on c-MYC signaling. As c-MYC is the first oncogene described to be regulated by BRD4 both in solid tumors and hematological malignancies^11,19^, we assessed whether JQ1 treatment would affect c-MYC levels by WB. The results showed that JQ1 obviously downregulated the protein expression of c-MYC in GC cells (Fig. 3a). To investigate the role of c-MYC in JQ1-triggered inhibition of GC cell migration and invasion, we transfected AGS and HGC27 cells with pcDNA3.1, a mock vector, and pcDNA3.1-c-MYC, a vector expressing wild-type c-MYC (Fig. 3b). We found that overexpression of c-MYC failed to antagonize the downregulation of migration and invasion of GC cells induced by JQ1 treatment (Fig. 3c, d). These data suggested that c-MYC signaling was not involved in JQ1-inducing inhibition of GC cell metastasis.

### JQ1 drives chromatin accessibility changes in GC cells and differentially accessible regions are highly enriched for RUNX2-binding motif

**Fig. 4:**
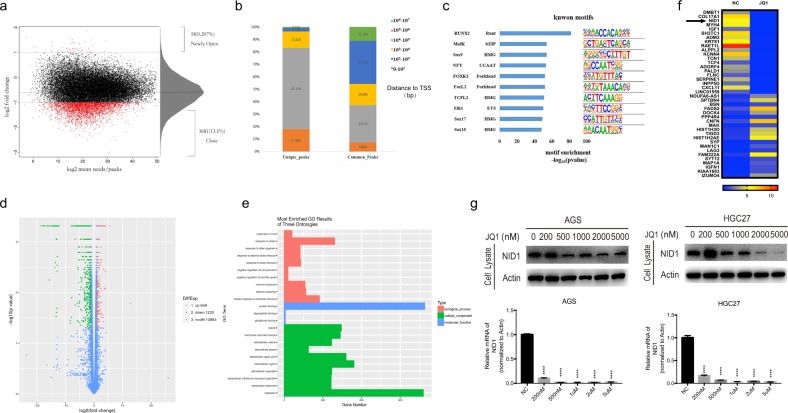
JQ1 drives chromatin accessibility changes in GC cells. **a** Overall chromatin accessibility changes of AGS cells following a 72 h treatment of JQ1 revealed by ATAC-seq analysis. **b** The location of the differentially accessible peaks were shown. **c** Motif-enrichment analysis of the differentially accessible sites. **d** The total number of differentially expressed genes of AGS cells after JQ1 treatment for 72 h revealed by RNA-seq analysis. **e** GO cluster analysis showed that the differentially expressed mRNAs were subjected to multiple GO terms. **f** The top 20 upregulated and top 20 downregulated genes after JQ1 treatment for 72 h demonstrated by RNA-seq analysis. **g** WB and qRT-PCR analysis of the mRNA and protein expression of NID1 in HGC27 and AGS cells treated with JQ1 (0, 200 nM, 500 nM, 1 μM, 2 μM, and 5 μM) for 72 h (mean ± SEM, ****p < 0.0001).

As c-MYC was not the key factor responsible for the suppression of GC cell metastasis induced by JQ1, we tried to seek for other molecular mechanisms underlying JQ1 treatment. As BET proteins are known as enhancers to activate multiple transcriptional targets by accumulating on specific histone lysine residues, we hypothesize that JQ1 may exert its anti-tumor effect by altering chromatin accessibility. Therefore, we performed ATAC-seq on AGS cells following 72 h treatment of JQ1 to assess the extent of chromatin accessibility changes. We found that ~13.1% of the accessible regions were more accessible (>2-fold) in the control group, whereas only 0.207% of the peaks were more accessible (>2-fold) in JQ1-treated cells, meaning that JQ1 obviously downregulated the chromatin accessibility in GC cells (Fig. 4a). The differentially accessible peaks (unique peaks) were mainly gene distal (>80%), located in regions more than 10^4^ bp to transcriptional start sites (TSS) (Fig. 4b), although relatively fewer differential peaks were located in promoter–proximal regions. Then we carried out motif-enrichment analysis of differentially accessible sites to identify potential drivers leading to the loss of chromatin accessibility caused by JQ1. As shown in Fig. 4c, the binding site for RUNX2 was the most highly enriched motif unique to controlled GC cells, suggesting that RUNX2 might play a crucial role in JQ1-induced changes of chromatin accessibility.

### JQ1 significantly reduces the expression of NID1 in GC cells

Then we performed RNA-seq analysis to determine the transcriptional target of JQ1 in GC cells. Using an false discovery rate of 0.05, we identified a total of 1880 differentially expressed genes with JQ1 treatment, of which 1232 were downregulated and 648 were upregulated (Fig. 4d). Moreover, Gene Ontology (GO) cluster analysis showed that the differentially expressed mRNAs were subjected to multiple GO terms, among which were the top 23 enriched terms including protein binding, cytoplasm, extracellular region, membrane-bounded vesicle, and response to stress (Fig. 4e). Among the 40 mostly dysregulated genes upon JQ1 treatment, we chose *NID1* as the target gene, because *NID1* has been reported to promote cancer metastasis in various cancer types^20^ (Fig. 4f). Besides, NID1 belonged to the most enriched GO term, protein binding. More importantly, we performed combinational analysis of the ATAC-seq and RNA-seq data, and discovered that of the top 40 dysregulated genes, *NID1* was the only gene associated with the nearest differentially accessible sites (chr1:235998701:236000920_intron). However, RUNX2 expression did not change significantly upon JQ1 treatment from RNA-seq analysis.

Then we assessed the expression of NID1 upon JQ1 treatment in AGS and HGC27 cells using WB and quantitative reverse-transcription real-time PCR (qRT-PCR), and confirmed that JQ1 significantly decreased both the mRNA and protein expression of NID1 in a seemingly dose-dependent manner in GC cells, consistent with the findings from RNA-seq data (Fig. 4g).

### JQ1 suppresses GC cell metastasis via mediating RUNX2/NID1 signaling in vitro

**Fig. 5:**
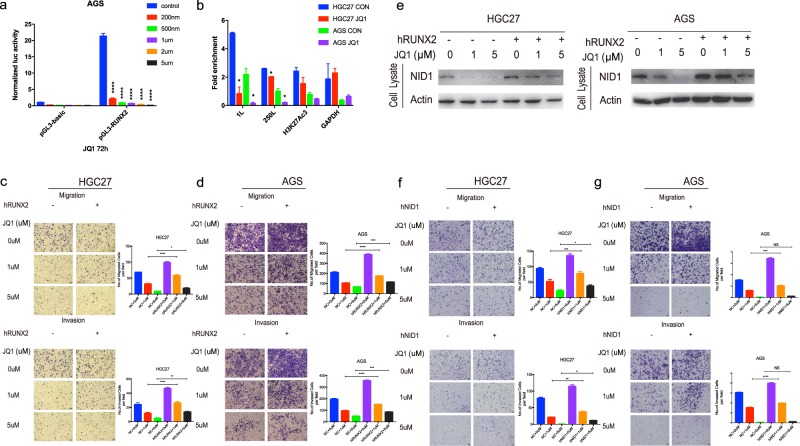
JQ1 suppresses GC cell metastasis via mediating RUNX2/NID1 signaling in vitro. **a** Luciferase reporter gene assay showed that the transcriptional activity of RUNX2 promoter in AGS cells was repressed by JQ1 (0, 200 nM, 500 nM, 1 μM, 2 μM, and 5 μM) (mean ± SEM, ****p < 0.0001). **b** ChIP-qRT-PCR analysis of BRD4 occupancy of the *RUNX2* gene in AGS cells after JQ1 (0, 1 μM) treatment for 72 h (mean ± SEM, *p < 0.05). **c**, **d** RUNX2 overexpression significantly antagonized the downregulation of the migration and invasion induced by JQ1 in HGC27 and AGS cells. Representative images of the migrated and invaded GC cells in each group were shown on the left. Cells that migrated and invaded through the pores of transwell plates were counted in five random fields and were reported on the right (mmean ± SEM, *p < 0.05, **p < 0.01, ***p < 0.001, ****p < 0.0001). **e** RUNX2 overexpression significantly attenuated the downregulation of NID1 expression induced by JQ1 in HGC27 and AGS cells detected by WB analysis. **f**, **g** NID1 overexpression significantly repressed the downregulation of the migration and invasion induced by JQ1 in HGC27 and AGS cells. Representative images of the migrated and invaded GC cells in each group were shown on the left. Cells that migrated and invaded through the pores of transwell plates were counted in five random fields and were reported on the right (mean ± SEM, *p < 0.05, **p < 0.01, ***p < 0.001, ****p < 0.0001).

Based on the ATAC-seq and RNA-seq data we analyzed, we next tried to explore the role of RUNX2 and NID1 in JQ1-inducing inhibition of GC cell metastasis. We hypothesized that JQ1 might affect the transcriptional activity of RUNX2 promoter without altering RUNX2 expression. To confirm this, we performed a luciferase reporter gene assay, demonstrating that JQ1 reduced the luciferase activity of RUNX2 promoter in a dose-dependent manner in AGS (Fig. 5a). Next, we moved on to chromatin immunoprecipitation (ChIP) assays followed by qRT-PCR analysis to determine whether BRD4 inhibition was the cause of RUNX2 inactivation by using BRD4 antibody in AGS and HGC27 cells. Several regions of interest were chosen, including one RUNX2 TSS (+1L), one upstream promoter region (−250L), and one H3K27Ac histone acetylation site (H3K27Ac-3). ChIP results showed that the enrichment of two regions, +1L and −250L, significantly diminished in JQ1-treated cells compared with that in control cells (Fig. 5b).

We then overexpressed RUNX2 in AGS and HGC27 cells with pcDNA3.1-RUNX2 plasmids and found that RUNX2 overexpression significantly enhanced JQ1-inhibiting migration and invasion in both GC cells (Fig. 5c, d). Moreover, WB demonstrated that overexpression of RUNX2 upregulated the protein expression of NID1 in GC cells. In addition, JQ1-inducing downregulation of NID1 protein expression was reversed by RUNX2 overexpression at both the concentrations, indicating NID1 as a downstream target of RUNX2 (Fig. 5e).

Next, we transfected AGS and HGC27 cells with pcDNA3.1-NID1, a vector expressing wild-type NID1, and the mock vector, to investigate whether NID1 was a crucial mediator for JQ1-suppressing GC metastasis. Transwell assays showed that overexpression of NID1 obviously attenuated the downregulation of migration and invasion induced by JQ1 (Fig. 5f, g). These data suggested that RUNX2/NID1 signaling was responsible for JQ1-suppressing GC cell metastasis in vitro.

### NID1 promotes the migration and invasion of GC cells partially through EMT process in vitro

**Fig. 6:**
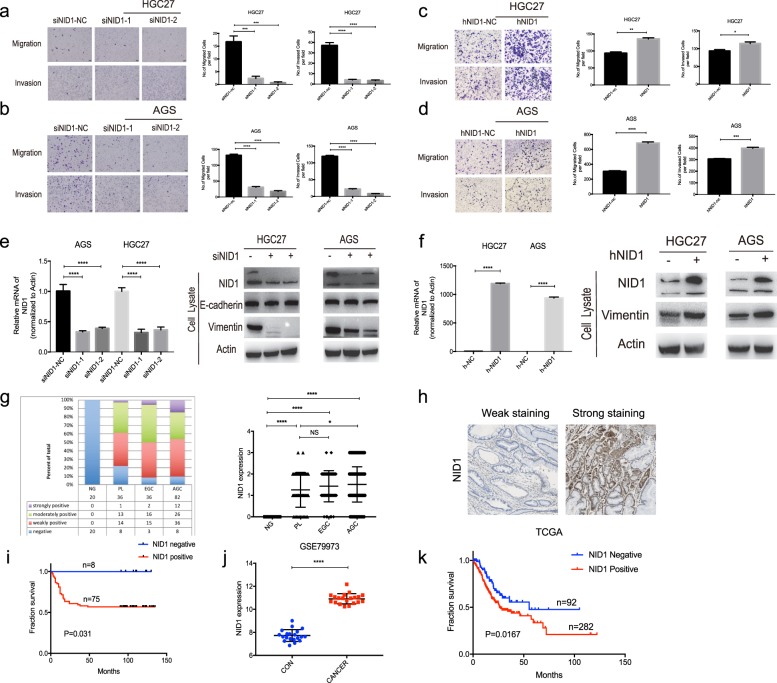
NID1 promotes the migration and invasion of GC cells partially through EMT process in vitro and overexpression of NID1 in GC tissues is associated with poor survival outcome of the patients. **a**, **b** Knockdown of NID1 impeded the migration and invasion of HGC27 and AGS cells. **c**, **d** Overexpression of NID1 increased the migration and invasion of HGC27 and AGS cells. For Fig. 6a–d, representative images of the migrated and invaded GC cells in each group were shown on the left. Cells that migrated and invaded through the pores of transwell plates were counted in five random fields and were reported on the right (mean ± SEM, *p < 0.05, **p < 0.01, ***p < 0.001, ****p < 0.0001). e Knockdown of NID1 inhibited the protein expression of Vimentin in HGC27 and AGS cells shown by WB analysis. **f** Overexpression of NID1 enhanced the protein expression of Vimentin in HGC27 and AGS cells indicated by WB analysis. **g** Differential expression of NID1 in patients with AGC, EGC, and PL compared with NG (mean ± SEM, NS > 0.05, *p < 0.05, ****p < 0.0001). **h** Immunohistochemistry staining showing weak expression and strong expression of NID1 in the tumor tissues from two patients with AGC. **i** Kaplan–Meier curves comparing overall survival in AGC patients with negative and positive expression of NID1 (*n* = 82; *P* = 0.031, log-rank test). **j** GEPIA (Gene Expression Profiling Interactive Analysis) data from the GEO databases (dataset 79,973) demonstrates elevated mRNA levels of NID1 in GC, compared with non-tumor tissues (mean ± SEM, ****p < 0.0001). **k** Kaplan–Meier curves comparing overall survival in GC patients from TCGA dataset (*n* = 374) with negative (*n* = 92) and positive (*n* = 282) expression of NID1 (*n* = 82; *P* = 0.0167, log-rank test).

NID1 has been proved to play a key role in promoting the motility and invasiveness of ovarian cancer cells via partial EMT process^20^. To investigate whether NID1 regulates GC cell metastasis, we introduced two small interfering RNAs (siRNAs) targeting NID1 into AGS and HGC27 cells. We found that NID1 silencing resulted in a significant downregulation of migratory and invasive GC cells compared with scrambled control (Fig. 6a, b), although NID1 overexpression led to obvious increase of GC cell migration and invasion indicated by transwell assays (Fig. 6c, d).

Then we asked whether NID1 could possibly regulate EMT process in GC cells. Figure 6e, f showed that NID1 depletion led to a decrease of the protein expression of Vimentin, a mesenchymal marker; however, the expression of the epithelial marker E-cadherin did not change after NID1 knockdown. Conversely, NID1 overexpression obviously enhanced the expression of Vimentin in both AGS and HGC27 cells. Taken together, NID1 promoted GC cell migration and invasion partially through EMT process in vitro.

### NID1 is overexpressed in GC tissues and associated with poor survival outcome of GC patients

As NID1 was a crucial regulator in GC cell metastasis, we decided to unveil the association between NID1 expression and the clinicopathologic characteristics of GC patients. Immunohistochemical staining of NID1 antibody was performed in gastric tissue samples from the same cohort as BRD4, including 20 NG, 36 PL, 36 EGC, and 82 AGC patients (Fig. 6h). All the 20 NG samples stained negative for NID1 antibody. Among the 36 PL samples, 77.8% of the patients exhibited weakly to strongly positive staining for NID1, whereas the positive rate in EGC patients turned out to be 91.7%. In addition, 92.0% of the AGC patients stained positive for NID1. Statistical analysis indicated that NID1 expression in PL, EGC, and AGC tissues was significantly higher than that in NG tissues, whereas NID1 expression in AGC was obviously higher than in PL, suggesting that NID1 might play an important role in the malignant progression of GC (Fig. 6g). What’s more, KM survival analysis showed that positive expression of NID1 was significantly associated with a poor survival outcome in these patients (*P* = 0.031) (Fig. 6i). In addition, we analyzed the mRNA expression of NID1 in GEO GSE79973 dataset and found that NID1 expression was significantly higher in GC compared with non-tumor gastric tissues (Fig. 6j). Besides, the KM survival analysis from The Cancer Genome Atlas dataset showed that positive mRNA expression of NID1 also predicted worse survival outcome compared with negative expression (Fig. 6k). Therefore, these data suggested that NID1 protein and mRNA levels were greatly increased in human GC, and served as a potential prognostic biomarker for GC patients.

### JQ1 effectively inhibits the proliferation of GC in vivo

**Fig. 7:**
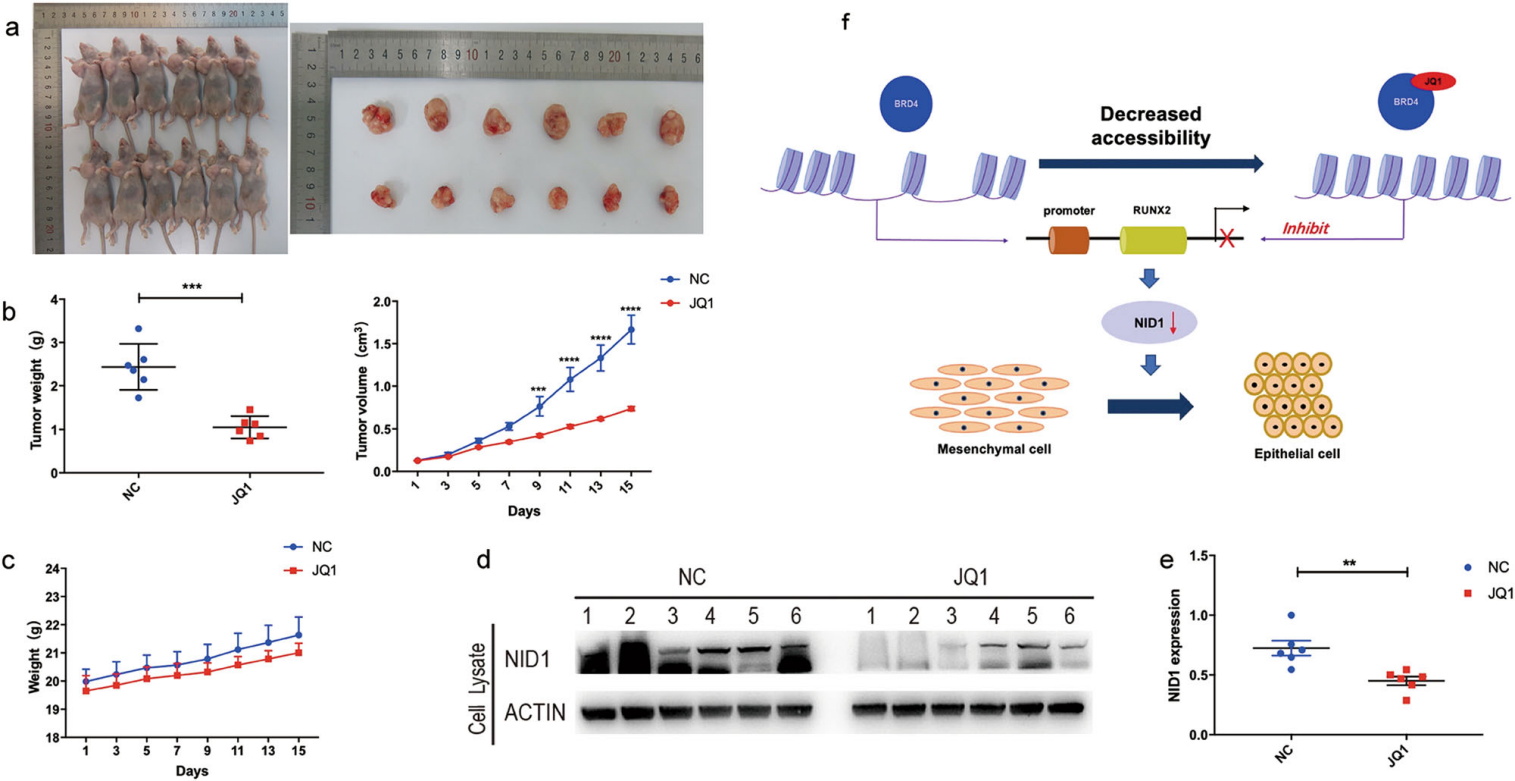
JQ1 effectively inhibits the proliferation of GC in vivo. **a** Twelve mice were divided into two groups and were treated with DMSO and JQ1, respectively. All mice and resected tumors were photographed after killing. **b** The weights and volumes of resected tumors from each mouse were recorded. **c** The body weights of each mouse were recorded. **d** The protein expression of NID1 of the resected tumors revealed by WB analysis. **e** The mRNA expression of NID1 of the resected tumors revealed by qRT-PCR analysis (mean ± SEM, **p < 0.01). **f** Schematic model for how JQ1 regulated the RUNX2/NID1 signaling via altering **c**hromatin accessibility in GC cells.

To further validate our findings, the anti-tumor effects of JQ1 were evaluated in vivo using a xenograft mouse model transplanted with HGC27 cells subcutaneously. Twelve mice were divided into two groups: the NC group and the JQ1-treating group. After 2 weeks of JQ1 treatment, we observed that the volumes and weights of the tumors from the JQ1-treating group were significantly decreased compared with that in the NC group (Fig. 7a, b). However, there were no obvious differences regarding body weights of the mice between the two groups (Fig. 7c). Then, total protein and mRNA were extracted from the fresh tumors. WB analysis showed that the NID1 protein expression was significantly downregulated in JQ1-treating group compared with NC group (Fig. 7d). In addition, qRT-PCR results demonstrated a significant decrease in NID1 mRNA expression after JQ1 treatment (Fig. 7e). These findings indicated that JQ1 suppressed GC tumor proliferation via inhibiting NID1 signaling in vivo.

## Discussion

Despite an overall decrease in morbidity over the past decades, GC is still one of the main causes of cancer-related deaths in China^21^. Treatment of AGC remains a major clinical challenge, especially for patients with metastases. Epigenetic mechanisms, especially chromatin alterations, are involved in the development of all types of cancers. An accumulation of studies has highlighted the role of histone modifications, e.g., histone methylation and acetylation, in GC progression and metastasis. Thus, it is worthy of developing epigenetic inhibitor compounds targeting these modifications for treating GC patients.

Members of BET family (BRD2, BRD3, BRD4, and BRDT) are “readers” of acetyl-lysine residues and play a key role in transcriptional elongation^22,23^. BET proteins have been identified as crucial regulators in oncogenic transcriptional programs for various cancers^22^, including GC. Ba et al.^24^ reported that BRD4 could promote the proliferation of GC cells via activating c-MYC signaling. Although Dong et al.^18^ described that inhibition of BRD4 promoted cellular senescence and repressed GC cell proliferation via modulating E2F/miR-106b/p21 axis. Our research also showed that BRD4 was highly expressed in AGC tissues, and predicted poor survival outcome in AGC patients, suggesting that BRD4 played a crucial role in promoting GC development. These data suggest that BRD4 is a promising therapeutic target for GC patients.

Until now, multiple BET inhibitors have been developed, among which JQ1 is a noteworthy anticancer drug that permeates into the cell membrane and has a structure that mimics acetyl-lysine, allowing it to bind to the acetyl-lysine pocket owned by bromodomain proteins^13^. Although JQ1 also inhibits other bromodomain proteins such as BRD2 and BRD3, it has been demonstrated that JQ1 is more specific to BRD4 than other bromodomain proteins, according to previous studies^11,13,25^. JQ1 was first reported to inhibit the proliferation of leukemia cells both in vivo and in vitro through downregulating c-MYC signaling^10^. Afterwards, more and more studies have focused on the anti-cancer effects of JQ1 in solid tumors, and found that JQ1 suppressed tumor growth via transcriptionally inactivating multiple oncogenic pathways other than c-MYC, e.g., YAP, Akt, and NOTCH3, in various cancer types including pancreatic cancer, colorectal cancer, prostate cancer, and breast cancer^26–28^. However in some circumstances, the cancer cells evade the apoptosis induced by JQ1 and fail to respond to JQ1 treatment, causing the delayed application of JQ1 clinically in cancer patients^29,30^. In addition, Montenegro et al.^21^ reported that compared with GC cells derived from Brazilian patients, those from Asian patients were more resistant to JQ1 treatment. Therefore, it is urgent for us to seek deeply into the precise molecular mechanisms underlying JQ1 treatment in GC cells.

Here, in our study, we showed that JQ1 reduced cell proliferation, induced cell apoptosis, and inhibited cell metastasis independent of c-MYC signaling both in vitro and in vivo, confirming the chemotherapeutic effects of JQ1 in GC cells. Then we applied ATAC-seq to assess the changes of chromatin accessibility of GC cells after JQ1 treatment, demonstrating that JQ1 generally closed 13.1% of the accessible regions in AGS cells, leading to a global inactivation of downstream TFs. The motif-enrichment analysis implicated that RUNX2 was highly enriched in the opened regions of the controlled cells compared with JQ1-treating cells. RUNX2 was initially regarded as a promoter of osteoblast differentiation and was later proved to regulate the development of various tumors^31–33^. Guo et al.^34^ indicated that RUNX2 was highly expressed in human GC tissues and promoted the metastasis of GC by transcriptionally upregulating CXCR4 signaling, verifying the correlation between RUNX2 and GC progression. In this research, ChIP and luciferase reporter gene assays both elucidated that JQ1 significantly inhibited the transcriptional activity of RUNX2 signaling, nevertheless failing to alter the expression of RUNX2. Moreover, rescue experiments indicated that RUNX2 was responsible for the repression of GC cell migration and invasion induced by JQ1.

We also performed RNA-seq to analyze how JQ1 influence the gene expression programs in GC cells. Combinational analysis of the ATAC-seq and RNA-seq data indicated NID1 as a downstream target of JQ1 treatment, which was correlated with the differentially accessible sites. As a secreted pro-metastatic protein, NID1 has been reported to promote lung metastasis of breast cancer and melanoma^35^. Zhou et al.^20^ also showed that NID1 promoted EMT of ovarian cancer cells through activating extracellular-signal-regulated kinase/mitogen-activated protein kinase signaling. Here, in our study, JQ1 obviously reduced the mRNA and protein expression of NID1 in all GC cells and NID1 was necessary for JQ1-inducing suppression of GC cell migration and invasion validated by rescue experiments. Besides, RUNX2 overexpression rescued the decreased expression of NID1 caused by JQ1, confirming NID1 as a downstream target of RUNX2. What’s more, we demonstrated that NID1 facilitated GC cell migration and invasion in vitro and high expression of NID1 correlated with poor survival outcome in GC patients, highlighting the role of NID1 in GC progression.

In conclusion, as a BET inhibitor, JQ1 inhibited proliferation and metastasis of human GC cells through inactivating RUNX2/NID1 signaling (Fig. 7f). We also found that JQ1 caused globally site-specific chromatin remodeling in the genome of GC cells, with more sites losing accessibility. This study offered new insights into the molecular mechanisms of JQ1 treatment in GC cells and provided novel approaches for progressive GC therapy. In addition, NID1 also worked as a potentially novel therapeutic target for progressive GC patients.

## Materials and methods

### Cell culture

The cells were maintained at 37 °C with 5% CO_2_. Three human GC cell lines were used: HGC27, AGS, and MKN45 (The Institute of Biochemistry and Cell Biology, Shanghai Institutes for Biological Sciences, Chinese Academy of Sciences, Shanghai, China). All cell lines were authenticated by Short Tandem Repeats profiling and tested for mycoplasma contamination. The cells were maintained in RPMI-1640 medium (Invitrogen, Waltham, MA, USA) containing 10% fetal bovine serum (FBS; Biological Industries, Cromwell, CT, USA), penicillin (Invitrogen) (100 U/ml), and streptomycin (Invitrogen) (100 U/ml). The cells were used for experiments after they reached to 70–80% confluence.

### ATAC-seq

ATAC-seq was performed as described^36^. Fifty thousand cells were collected and centrifuged. The cell pellets were resuspended in lysis buffer (10 mM Tris-HCl, pH 7.5, 10 mM NaCl, 3 mM MgCl_2_, and 0.1% IGEPAL CA-630) and immediately centrifuged at 500 × *g* at 4 °C for 10 min. The supernantant was removed and nuclei resuspended in reaction buffer (1× TD buffer with 2.5 μl of Tn5 transposase in 50 μl total) for 30 min at 37 °C. DNA was purified using the MinElute Reaction Cleanup kit (QIAGEN). Libraries were amplified with Illumina Nextera sequencing primers and quantified by qRT-PCR against PhiX standard. Finally, the high-quality DNA libraries were sequenced on the Illumina HiSeq2000. Data were analyzed following the previously established protocol^37^. Peak-related genes were subjected to GO and Kyoto Encyclopedia of Genes and Genomes annotation, and Homer-motif analysis.

### RNA-sequencing

AGS cells plated in 10 cm dishes were treated with dimethyl sulfoxide (DMSO) or JQ1 for 3 days, respectively. Then cells were washed by phosphate-buffered saline (PBS) for three times and then lysed by Trizol at room temperature. A total amount of 3 mg RNA per sample was used as input material for the RNA sample preparations. Sequencing libraries were generated using NEBNext UltraTM RNA Library Prep for Illumina (NEB, USA) following the manufacturer’s recommendations, and index codes were added to attribute sequences to each sample. The clustering of the index-coded samples was performed on a cBot Cluster Generation System using TruSeq PE Cluster Kit v3-cBot-HS (Illumia), according to the manufacturer’s instructions. After cluster generation, the library preparations were sequenced on an Illumina platform and 125 bp/150 bp paired-end reads were generated.

### siRNA transfection and plasmid transfection

The matching scrambled control siRNAs and chemically synthesized NID1 siRNAs were purchased from Hanbio Biotechnology Company (Shanghai, China). Their corresponding sequences are listed in Supplementary Table 1. The vectors expressing NID1 (pcDNA3.1‐NID1) and RUNX2 (pcDNA3.1‐RUNX2), as well as the blank pcDNA3.1 vector were purchased from Hanbio Biotechnology Company (Shanghai, China). The pGL3-basic, pRL-TK, and pGL3-RUNX2 luciferase reporter vectors were also purchased from Hanbio Biotechnology Company (Shanghai, China). Transient transfection was performed using Lipofectamine RNAiMax Transfection Reagent (Invitrogen, 13778150) for siRNAs and GeneJuice Transfection Reagent (Merck Millipore, 70967) for the plasmids, according to the established protocols from the manufacturers separately.

### Reagents and antibodies

JQ1, the BET inhibitor, was purchased from Selleckchem (Houston, TX, USA). We utilized the following antibodies in our research: anti-BRD4 (ab128874), anti-NID1 (ab133686), anti-c-MYC (ab32072), anti-E-cadherin (ab231303), and anti-vimentin (ab8978) antibodies from Abcam (Cambridge, MA, USA); anti-BRD4 (13440S), anti-E-cadherin (3195S), anti-Snail (3879S), anti-caspase-3 (14220), and anti-pro-caspase-3 (9661) antibodies from Cell Signaling Technology (Danvers, MA, USA); anti‐RUNX2 (sc-390715) antibody from Santa Cruz Biotechnology (Santa Cruz, CA, USA); the anti‐vimentin (10366-1-AP) antibody from ProteinTech Group (Rosemont, IL, USA); the anti-NID1 (AF2570) antibody from R&D Systems (Minneapolis, MN, USA); and the anti‐β‐actin (A5441) antibody from Sigma‐Aldrich (St Louis, MO, USA).

### Luciferase report assay

A total of 1 × 10^5^ AGS cells were seeded into 48-well plates for 24 h before transfection. pGL3-RUNX2 plasmid and pGL3-basic vector were transfected into the cells using GeneJuice Transfection Reagent (Merck millipore, 70967), according to the manufacturer’s protocol. The pGL3-RUNX2 luciferase reporter vector was constructed by subcloning 2000 bp fragments of the RUNX2 proximal promoter into the pGL3 basic vector, respectively. After 24 h of transfection, cells were treated with JQ1 for 72 h.Then the cells were collected and the luciferase activity of RUNX2 promoter was detected using the Dual-Luciferase Reporter System (Promega). The CMV-RenillaLuc plasmid was used as an internal control for transcription efficiency. Each experiment was repeated for three times.

### Cell viability assay

GC cells were seeded into 96-well plates (3 × 10^3^ cells/well). Different concentrations of JQ1 (0, 200 nM, 500 nM, 1 μM, 2 μM, 5 μM) or 0.1% DMSO were added at indicated times. A total of 10 µl CCK8 solutions (Dojindo, Minato-ku, Tokyo, Japan) was added to each well. Next, the cells were incubated at 37 °C for 1 h. Absorbance was recorded at 450 nm.

### Quantitative reverse-transcription real-time PCR

The total RNA of the cells was extracted with Trizol Reagent (Invitrogen Life Technologies) and subsequently reverse‐transcribed using the PrimeScript RT Master Mix, according to the manufacturer’s instructions. qRT‐PCR was carried out with the 7500 Real‐time PCR System (Applied Biosystems) using SYBR Premix Ex Taq reagents. The PCR cycling conditions were 40 cycles of 5 s at 95 °C and 32–34 s at 60 °C. All data were normalized to the human β‐actin. The sequences of specific primers are listed in Supplementary Table 2 and the primers were constructed in GENEWIZ (Suzhou, China). Fold induction was calculated using the formula 2 − ΔΔCt. The data represented were based on three independent experiments.

### Migration and invasion assays

Cellular motility and invasive abilities were determined using Transwell (Corning Life Sciences, Bedford, MA, USA) and Matrigel invasion (BD Biosciences, San Jose, CA, USA), respectively. For the transwell migration and invasion assay, HGC27 cells and AGS cells suspended in 500 μL of RPMI-1640 were seeded in the upper chamber at a density of 5 × 10^4^ cells/well and 2 × 10^5^ cells/well, respectively. Then we added 750 μL of RPMI‐1640 containing 15% FBS into the lower chamber. The cells in the upper chamber were removed after 24 h. Then the cells migrating through the membrane to the underside were fixed with methanol for 15 min and were subsequently stained with 0.5% crystal violet for 15 min. We counted the cell number in five separate fields using light microscopy at ×200 magnification. The average value of the migrating or invading cells were expressed as percentages. Each experiment was performed in replicate inserts and the average value was calculated from three independent experiments.

### Colony-formation assay

GC cells were counted and seeded into 6-well plates with 500 cells/well. After treatment with 0, 200 nM, 500 nM, and 1 μM JQ1 for 72 h, the drugs were removed and replaced with fresh medium for 14 days. The cells were fixed in methanol and colonies were counted after staining with 0.1% crystal violet in 25% methanol for 20 min. The results were presented as the average number of counted colonies per well under each condition. All the experiments were performed in triplicate and repeated three times independently.

### Apoptosis assay

Apoptosis was detected by flow cytometry using the Annexin V-fluorescein isothiocyanate Apoptosis Detection Kit (556, 547, BD Biosciences), following the manufacturer’s instructions.

### Western blotting

GC cells were lysed using RIPA buffer (150 mM NaCl, 50 mM Tris-HCl at pH 7.4, 1 mM EDTA, 0.1% SDS, 1% Triton X-100, 1% sodium deoxycholate, and 1% NP-40) mixed with a protease and phosphatase inhibitor cocktail (Roche Diagnostics GmbH, Mannheim, Germany) and phenylmethylsulfonyl fluoride (Biosharp, Hefei, China) for 15 min on ice. The proteins were subjected to WB according to standard protocols.

### Clinical specimens

One hundred and fifty-four tissue samples from patients of gastric PL/36, EGC/36, and AGC/82 were included in our study, with complete clinical information and available tissue blocks. In addition, 20 NG mucosa samples obtained from healthy volunteers were also enrolled in this study. Inclusion and exclusion criteria were described in detail in our previously published manuscripts^38^. Writing informed consent was acquired from both patients and healthy volunteers. The use of all tissue specimens in our study was approved by the Institutional Ethics Review Board of the affiliated Drum Tower Hospital of Nanjing University Medical School.

### Immunohistochemistry

Slides of the tumors were deparaffinized, blocked, and incubated at 4 °C overnight with primary antibodies, followed by secondary antibodies at room temperature for 1 h. BRD4 (ab128874, Abcam), RUNX2 (sc-390715, Santa Cruz), and NID1 (AF2570, R&D) antibodies were used.

### Immunofluorescence

GC cells from each group were seeded in 24-well plates at a density of 3 × 10^4^ cells per well. Twenty-four hours after seeding, cells were fixed with 4% paraformaldehyde for 15 min at room temperature and permeabilized with 0.5% Triton X-100 for 30 min. After blocking with 5% bovine serum albumin for 1 h at room temperature, cells were incubated with E-cadherin or vimentin antibodies overnight at 4 °C. Cells were washed three times and then incubated with secondary antibodies for 1 h at room temperature in the dark. Then, cells were incubated with DAPI (D9542, Sigma) for 5 min. Fluorescent images were observed and analyzed with a Fluorescence microscope (Nikon Eclipse CI, Japan).

### Wound-healing assay

GC cells were plated into six-well plates at a concentration of 1 × 10^5^ cells per well. Cells reached ~90% confluence 72 h after JQ1 (0, 200 nM, 500 nM, 1 μM, 2 μM, 5 μM) treatment. The cell monolayer was scratched with a yellow 200 µL pipette tip to generate a linear scratch wound. The culture medium was replaced with FBS-free RPMI-1640 medium. Cells were cultured for 24 h. Scratch-wound images at 0 and 24 h were captured using an Olympus IX71 microscope and wound-healing ability was calculated based on the relative cell-free area normalized to that in the 0 h image

### Chromatin Immunoprecipitation

GC cells (2 × 10^6^) plated in 10 cm dish were treated with DMSO or JQ1 at 1 μM for 3 days, respectively. Following 3 days of incubation, cells were fixed with 1% paraformaldehyde for 10 min at room temperature. Cells were then quenched with glycine for 5 min at room temperature. ChIP was performed using Magna ChIP A/G kit (Millipore) according to the manufacturer’s protocol. The cell lysate was prepared and equal amounts of DNA isolated from either DMSO- or JQ1-treated samples were immunoprecipitated using antibodies against BRD4 (Abcam) and IgG (Millipore) overnight at 4 °C. The DNA was eluted and subjected to quantitative PCR using SYBR Premix Ex Taq reagents for RUNX2. ChIP primer sets for RUNX2 promoter regions are listed in Supplementary Table 3.

### GC xenograft model

Female nude mice were subcutaneously injected in the axillary with 100 μL PBS or 1 × 10^7^ HGC27 cells suspended in 100 μL Matrigel. The mice were allowed to recover after the injections and monitored for 3 weeks. Tumors were measured using a caliper and once the majority of tumors reached a threshold size of 1 cm, i.p. injections of DMSO or JQ1 were initiated. The i.p. injections of 50 mg/kg JQ1 in 200 μL DMSO were administered once a day. After 2 weeks of treatment, or if the mice met the humane endpoint criteria, they were killed. Half of the tumor tissues were collected, fixed with 10% neutral formaldehyde, embedded in paraffin, sectioned, and stained with hematoxylin and eosin (HE). The remaining tumor tissues were frozen in liquid nitrogen and then stored at −80 °C for subsequent analyses.

### In vivo metastasis assay

For the peritoneal dissemination assay, 100 μL PBS or 2 × 10^6^ HGC27 cells suspended in 100 μL PBS were injected into the abdominal cavity of twelve 5-week-old male nude mice. The mice were allowed to recover after the injections and monitored for 3 weeks. Then the mice were divided into two groups and JQ1 (50 mg/kg) or DMSO was injected into the abdominal cavity once a day. Two weeks later, the mice were killed and the nodules were observed and counted. Tissues were collected, fixed with 10% neutral formaldehyde, sectioned, and stained with HE. We created the different groups of mice without any specific randomization scheme. All procedures involving animals and their care were conducted in conformity with the institutional guidelines, which are in compliance with national and international laws.

### Statistics

Each experiment was repeated at a minimum of three times. The data are expressed as mean ± SEM. Single factor analysis of variance tests was used for multiple comparisons and Student’s *t*-test was used for comparisons between two groups (*p < 0.05, **p < 0.01, ***p < 0.001, and ****p < 0.0001). SPSS 19.0 (SPSS, Inc., Chicago, IL, USA) and GraphPad Prism 7.0 (GraphPad Software, La Jolla, CA, USA) were used for the statistical analysis.

## Supplementary information

Supplementary Information

Supplementary Figure 1

## References

[1] Catalano V (2005). Gastric cancer. Crit. Rev. Oncol. Hematol..

[2] Gallo A, Cha C (2006). Updates on esophageal and gastric cancers. World J. Gastroenterol..

[3] Corces MR (2016). Lineage-specific and single-cell chromatin accessibility charts human hematopoiesis and leukemia evolution. Nat. Genet..

[4] Atlasi Y, Stunnenberg HG (2017). The interplay of epigenetic marks during stem cell differentiation and development. Nat. Rev. Genet..

[5] Stadhouders R (2018). Transcription factors orchestrate dynamic interplay between genome topology and gene regulation during cell reprogramming. Nat. Genet..

[6] Andreoli F, Barbosa AJ, Parenti MD, Del Rio A (2013). Modulation of epigenetic targets for anticancer therapy: clinicopathological relevance, structural data and drug discovery perspectives. Curr. Pharm. Des..

[7] Shi J, Vakoc CR (2014). The mechanisms behind the therapeutic activity of BET bromodomain inhibition. Mol. Cell.

[8] Lee DH (2015). Synergistic effect of JQ1 and rapamycin for treatment of human osteosarcoma. Int. J. Cancer.

[9] Kanno T (2014). BRD4 assists elongation of both coding and enhancer RNAs by interacting with acetylated histones. Nat. Struct. Mol. Biol..

[10] Zuber J (2011). RNAi screen identifies Brd4 as a therapeutic target in acute myeloid leukaemia. Nature.

[11] Delmore JE (2011). BET bromodomain inhibition as a therapeutic strategy to target c-Myc. Cell.

[12] Ott CJ (2012). BET bromodomain inhibition targets both c-Myc and IL7R in high-risk acute lymphoblastic leukemia. Blood.

[13] Filippakopoulos P (2010). Selective inhibition of BET bromodomains. Nature.

[14] Lockwood WW, Zejnullahu K, Bradner JE, Varmus H (2012). Sensitivity of human lung adenocarcinoma cell lines to targeted inhibition of BET epigenetic signaling proteins. Proc. Natl Acad. Sci. USA.

[15] Puissant A (2013). Targeting MYCN in neuroblastoma by BET bromodomain inhibition. Cancer Discov..

[16] Bandopadhayay P (2014). BET bromodomain inhibition of MYC-amplified medulloblastoma. Clin. Cancer Res..

[17] Dang CV (2012). MYC on the path to cancer. Cell.

[18] Dong X, Hu X, Chen J, Hu D, Chen LF (2018). BRD4 regulates cellular senescence in gastric cancer cells via E2F/miR-106b/p21 axis. Cell Death Dis..

[19] McCleland ML (2016). CCAT1 is an enhancer-templated RNA that predicts BET sensitivity in colorectal cancer. J. Clin. Invest..

[20] Zhou Y (2017). NID1, a new regulator of EMT required for metastasis and chemoresistance of ovarian cancer cells. Oncotarget.

[21] Montenegro RC (2016). BET inhibition as a new strategy for the treatment of gastric cancer. Oncotarget.

[22] Filippakopoulos P, Knapp S (2014). Targeting bromodomains: epigenetic readers of lysine acetylation. Nat. Rev. Drug Discov..

[23] Belkina AC, Denis GV (2012). BET domain co-regulators in obesity, inflammation and cancer. Nat. Rev. Cancer.

[24] Ba M (2018). BRD4 promotes gastric cancer progression through the transcriptional and epigenetic regulation of c-MYC. J. Cell Biochem..

[25] Shu S, Polyak K (2016). BET bromodomain proteins as cancer therapeutic targets. Cold Spring Harb. Symp. Quant. Biol..

[26] Liu C (2019). Suppression of YAP/TAZ-Notch1-NICD axis by bromodomain and extraterminal protein inhibition impairs liver regeneration. Theranostics.

[27] Wang J (2018). Targeting c-Myc: JQ1 as a promising option for c-Myc-amplified esophageal squamous cell carcinoma. Cancer Lett..

[28] Frank SB, Berger PL, Ljungman M, Miranti CK (2017). Human prostate luminal cell differentiation requires NOTCH3 induction by p38-MAPK and MYC. J. Cell Sci..

[29] Rathert P (2015). Transcriptional plasticity promotes primary and acquired resistance to BET inhibition. Nature.

[30] Shu S (2016). Response and resistance to BET bromodomain inhibitors in triple-negative breast cancer. Nature.

[31] Ducy P, Zhang R, Geoffroy V, Ridall AL, Karsenty G (1997). Osf2/Cbfa1: a transcriptional activator of osteoblast differentiation. Cell.

[32] Sancisi V (2017). RUNX2 expression in thyroid and breast cancer requires the cooperation of three non-redundant enhancers under the control of BRD4 and c-JUN. Nucleic Acids Res..

[33] Cohen-Solal KA, Boregowda RK, Lasfar A (2015). RUNX2 and the PI3K/AKT axis reciprocal activation as a driving force for tumor progression. Mol. Cancer.

[34] Guo ZJ (2016). Transcription factor RUNX2 up-regulates chemokine receptor CXCR4 to promote invasive and metastatic potentials of human gastric cancer. Oncotarget.

[35] Alečković M (2017). Identification of Nidogen 1 as a lung metastasis protein through secretome analysis. Genes Dev..

[36] Buenrostro JD, Giresi PG, Zaba LC, Chang HY, Greenleaf WJ (2013). Transposition of native chromatin for fast and sensitive epigenomic profiling of open chromatin, DNA-binding proteins and nucleosome position. Nat. Methods.

[37] Qu K (2017). Chromatin accessibility landscape of cutaneous T cell lymphoma and dynamic response to HDAC inhibitors. Cancer Cell..

[38] Zhang S (2017). Co-ordinated overexpression of SIRT1 and STAT3 is associated with poor survival outcome in gastric cancer patients. Oncotarget.

