# Supplementary material for: BET protein inhibitor JQ1 downregulates chromatin accessibility and suppresses metastasis of gastric cancer via inactivating RUNX2/NID1 signaling
Source: Oncogenesis. 2020 Mar 10;9(3):33. doi: 10.1038/s41389-020-0218-z (PMC7064486; doi:10.1038/s41389-020-0218-z)
Supplement: Supplementary file 1 — Supplementary Information [file 41389_2020_218_MOESM1_ESM.docx]

Supplementary Table 1

| **NID1-siRNA Alignments** | | | |
| --- | --- | --- | --- |
|  | Accession | Description | Sequence |
| **NID1-si-1** | XM_0115441952 | REDTTED：Homo sapiens nidogen 1(NID1) transcript variant X1, mRNA | 5’-CAACGGAGCUUAUAACAUAUU-3’ |
|  | NM_0025082 | Homo sapiens nidogen 1(NID1), mRNA |  |
| **NID1-si-2** | XM_0115441952 | REDTTED：Homo sapiens nidogen 1(NID1) transcript variant X1, mRNA | 5’-GGAAAUACCAUGAGGAAGAUU-3’ |
|  | NM_0025082 | Homo sapiens nidogen 1(NID1), mRNA |  |

Supplementary Table 2

| **Gene ID** | **Forward Primer Sequence**  **Reverse Primer Sequence** | **Note** | |
| --- | --- | --- | --- |
| **For quantitative reverse-Transcription real-time PCR（qRT-PCR）** | | | |
| **NID1** | 5’-GACTGACCTTCGATGCGTTC-3’  5’-CAAGATCGAGAGCAACCACG-3’ |  | |
| **β-actin** | 5’-CATGTACGTTGCTATCCAGGC-3’  5’-CTCCTTAATGTCACGCACGAT-3’ |  | |
| Supplementary Table 3 | | | |
| **For Chromatin Immunoprecipitation (ChIP)** | | | |
| **RUNX2**  **+1L** | 5’-TCACTACCAGCCACCGAGA-3’  5’-GGCTCACGTCGCTCATTT-3’ | | Transcription start site (TSS) |
| **RUNX2**  **-250L** | 5’-TACAGGAGTTTGGGCTCCTTC -3’  5’-CTTTTCCCCCTTGCTCTTTC -3’ | | 250 bp upstream of TSS |
| **RUNX2**  **H3KAc27-2** | 5’-TCAAGTGGACCAGGGTTTTG-3’  5’-CTCCGATGACCACTTTAGGA-3’ | | H3KAc27-2 histone Acetylation site 2 |
| **GAPDH** | 5’-CAGCAAGCGCACAAGAGGAA-3  5’-TCTACATGGCAACTGTGAGGAG-3 | | Loading control |

**Figure Legends**

**Supplementary Figure 1 The effects of JQ1 on cell proliferation, apoptosis, and migration of MKN45 cells.**

(a) The proliferation rates of MKN45 cells treated with JQ1 (0, 200nM, 500nM, 1uM, 2uM, & 5uM) for 72h determined by CCK8 assay.

(b) Flow cytometry analysis of the apoptotic cells treated with JQ1 (0, 200nM, 500nM, 1uM, 2uM, 5uM) for 72h using the Annexin Ⅴ/PI staining assay. Positive AnnexinⅤ cells were displayed as histogram. (Mean ± SEM, ****p < 0.0001 compared with control cells)

(c) The migratory capability of MKN45 cells treated with JQ1 (0, 200nM, 500nM, 1uM, 2uM, & 5uM) for 72h revealed by wound healing assay.
