# Supplementary figures and images for: BET protein inhibitor JQ1 downregulates chromatin accessibility and suppresses metastasis of gastric cancer via inactivating RUNX2/NID1 signaling
Source: Oncogenesis. 2020 Mar 10;9(3):33. doi: 10.1038/s41389-020-0218-z (PMC7064486; doi:10.1038/s41389-020-0218-z)

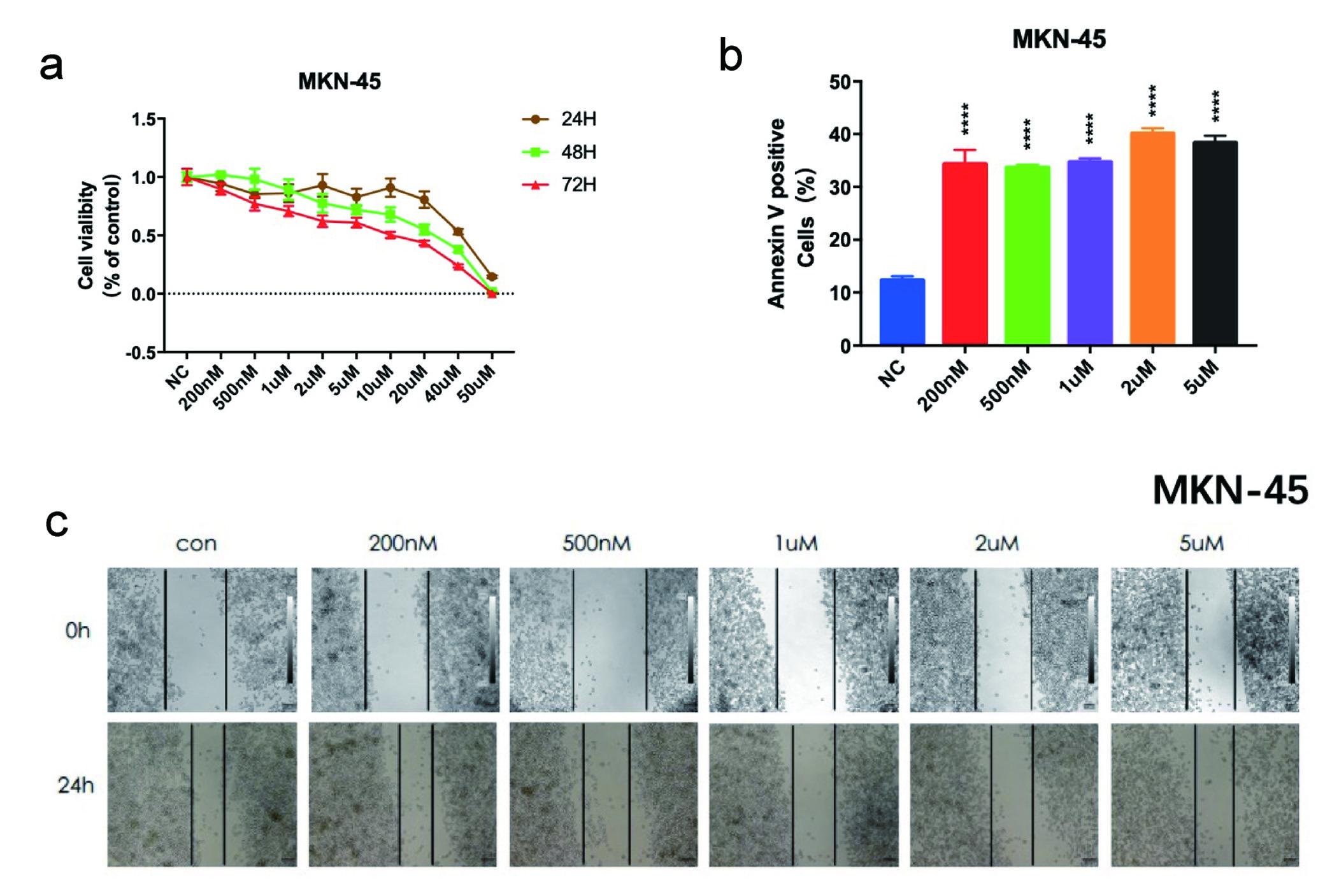

Supplement: Supplementary file 2 — Supplementary Figure 1 [file 41389_2020_218_MOESM2_ESM.jpg]
